# Supplementary material for: Identification of photoperiod-induced specific miRNAs in the adrenal glands of Sunite sheep (Ovis aries)
Source: Front Vet Sci. 2022 Jul 22;9:888207. doi: 10.3389/fvets.2022.888207 (PMC9354845; doi:10.3389/fvets.2022.888207)
Supplement: Supplementary file 1 [file Data_Sheet_1.ZIP › Supplementary file/miRDeep2 file.zip!/miRDeep2 file/Know_Structure/SP42A3/oar-mir-1185.pdf]

miRBase precursor	: oar-mir-1185
Total read count	: 105
oar-miR-1185-5p read count	: 36
oar-miR-1185-3p read count	: 69
remaining reads	: 0

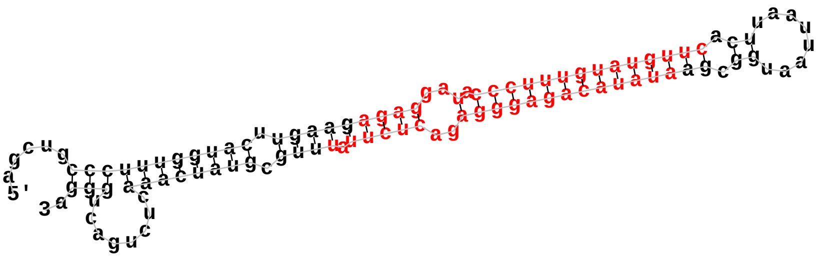

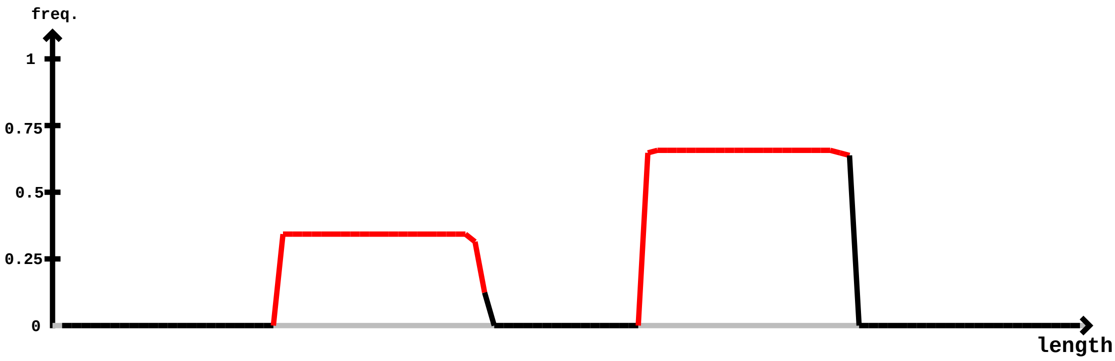


```
                      oar-miR-1185-5p
                                                                oar-miR-1185-3p
5'- agcugcccuuugguacuugaagagaggauacccuuuguauguucacuuaauuaauggcgaauauacagagggagacucuuauuugcguaucaaacucugacuggga  -3'   exp
    .....(((((((((((.(((((((((..(.((((((((((((((.((........)).)))))))))))))))..))))).)))).))))))))).......))).   reads   mm   sample
    ......................agaggauacccuuuguaugu................................................................   3       0    seq
    ......................agaggauacccuuuguauguu...............................................................   20      0    seq
    ......................agaggauacccuuuguauguuc..............................................................   13      0    seq
    ............................................................auauacagagggagacucuu..........................   1       0    seq
    ............................................................auauacagagggagacucuua.........................   1       0    seq
    ............................................................auauacagagggagacucuuau........................   66      0    seq
    .............................................................uauacagagggagacucuuau........................   1       0    seq
```
